# Role of αβ T Cell Depletion in Prevention of Graft versus Host Disease

**DOI:** 10.3390/biomedicines5030035

**Published:** 2017-06-26

**Authors:** Haitham Abdelhakim, Hisham Abdel-Azim, Ayman Saad

**Affiliations:** 1 Division of Hematology and Oncology, University of Kansas Cancer Center, Kansas City, KS 66205, USA; habdelhakim@kumc.edu; 2 Division of Hematology, Oncology and Blood and Marrow Transplantation, Children’s Hospital Los Angeles, University of Southern California Keck School of Medicine, Los Angeles, CA 90027, USA; habdelazim@chla.usc.edu; 3 Section of Blood and Marrow Transplantation and Cellular Therapy, University of Alabama at Birmingham, Brimingham, AL 35294, USA

**Keywords:** αβ T cells, haploidentical transplant, graft versus host disease

## Abstract

Graft versus host disease (GVHD) represents a major complication of allogeneic hematopoietic stem cell transplantation (allo HCT). Graft cellular manipulation has been used to mitigate the risk of GVHD. The αβ T cells are considered the primary culprit for causing GVHD therefore depletion of this T cell subset emerged as a promising cellular manipulation strategy to overcome the human leukocyte antigen (HLA) barrier of haploidentical (haplo) HCT. This approach is also being investigated in HLA-matched HCT. In several studies, αβ T cell depletion HCT has been performed without pharmacologic GVHD prophylaxis, thus unleashing favorable effect of donor’s natural killer cells (NK) and γδ T cells. This article will discuss the evolution of this method in clinical practice and the clinical outcome as described in different clinical trials.

## 1. Introduction

Allogeneic hematopoietic stem cell transplantation (allo HCT) is a treatment modality for a variety of malignant and non-malignant diseases [1]. Only about 30% of patients have an HLA-matched sibling and in about 16–75% (depending on ethnicity), an HLA-matched unrelated donor can be identified [1]. In the absence of an HLA-matched sibling or volunteer donor, alternative donor of hematopoietic stem cells (HSC), such as unrelated umbilical cord blood (UCB) [2,3,4,5] or haplo donor [6,7,8,9,10,11] can be utilized. The main advantages of UCB products are the immediate availability and low risk of graft versus host disease (GVHD) with mismatched products (immature lymphocyte content). The disadvantages are high cost (30,000–40,000 US dollars per unit), difficult finding of units with adequate HSC for adults (in particular for overweight/obese recipients), delayed engraftment and immune reconstitution, and lack of the donor lymphocyte infusion (DLI) option. On the other hand, the main advantages of haplo HSC products are the availability of donors in most cases (first degree relative either parent, sibling or a child), relatively lower cost, faster engraftment (compared to UCB), and availability of DLI option.

The initial successful haplo HCT relied primarily on T cell depletion to control the high risk of GVHD induced by the high HLA disparity in this setting [12,13]. This approach also allowed elimination of post-transplant immunosuppression therapy (IST) which may unleash T cell-mediated graft versus tumor (GVT) effect. This approach would also avert several side effects related to the use of IST such as high risk of posterior reversible encephalopathy syndrome which is more pronounced in sickle cell patients receiving calcineurin inhibitors [14]. T cell depletion techniques have been refined to selectively deplete αβ CD3+ cells that are believed to be involved in the pathogenesis of GVHD. With that approach, the minor population of γδ T cells and natural killer (NK) cells are spared and thus likely resulting in enhanced post-transplant immune recovery with less infection and perhaps less relapse risk. In this review, we will describe the clinical utility of αβ T cell depletion HCT outlining the evolution of this method, the technical method and clinical outcome.

## 2. Evolution of T Cell Depletion Methods

**Figure 1:**
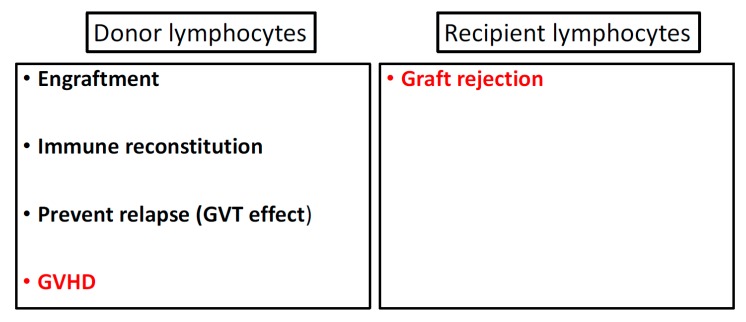
Immune balance between donor and recipient lymphocytes showing favorable (**black**) and unfavorable (**red**) effects played by each side. GVT = graft versus tumor.

While the T cell component of allo HSC product supports engraftment, and immune reconstitution and combats relapse (via GVT effect), they also induce GVHD (Figure 1). The separation of GVHD and GVT effect is an optimal target for an allo HCT platform. Peripheral blood stem cells (PBSC) product has 1-log more T cell dose compared to bone marrow (BM) and has indeed been associated with higher risk of GVHD [15,16]. The role of T cells as mediator of GVT was illustrated in the seminal study by the Center for International Blood and Marrow Transplant Research (CIBMTR) that analyzed data of 2254 patients and showed higher risk of relapse of hematological malignancies among patients who received T cell depleted graft [17]. Since then, escalating doses of DLI have been utilized in an attempt to treat relapsed neoplastic diseases, in particular, chronic myeloid leukemia, with promising success [18]. Moreover, this observation prompted interest in post-transplant add-back T cell therapy to enhance post-transplant immune reconstitution and control relapse risk. In this approach, 1-2 add-back T cell doses of 1 × 10^7^ cells/kg was infused post-transplant before day +100 [19,20,21,22]. A long-term (4 year follow up) result using this approach showed comparable outcome to T cell replete allo HCT with rates of grade II-IV aGVHD and cGVHD of 39% and 36% respectively and relapse rate of 40% [22]. In contrast, pan T cell depletion was used to overcome the HLA mismatching barrier of haplo HCT after the initial use of T cell replete haplo HCT resulted in prohibitively high risk of graft failure and GVHD [23,24,25]. Although in general, pan T cell depletion (<1 × 10^5^ CD3+ cells/kg) has been successful in reducing risk of GVHD [26,27], it has been associated with slow immune recovery and high rate of post-HCT infection [13]. The outcome of this approach has improved with using “mega-dose” of G-CSF-mobilized PBSC, as reported by the Perugia group with a cumulative incidence of aGVHD (grade II-IV) and cGVHD below10% [26]. The impact of the CD34+ stem cell dose on the outcome of T cell depleted haplo HCT is illustrated in a study (*n* = 127) by The Acute Leukemia and Pediatric Working Parties of the European Blood and Marrow Transplantation (EBMT) Group that showed improved DFS among patients who received CD34+ stem cell dose greater than 12 × 10^6^/kg [7]. A modified approach of T cell depletion, CD3+/CD19+ cell depletion has also been used to eliminate the increased risk of Epstein Barr virus (EBV) reactivation which was noted in initial T cell depletion studies [28,29]. This additional B cell depletion is also thought to likely reduce the risk of cGVHD which is believed to be primarily B cell-mediated. Selective depletion of CD8+ T cell has also been attempted hypothesizing that this T cell subset is the effector mediator of the tissue damage of GVHD. However, despite initial promising results, this method failed to improve the rate of GVHD in a phase II clinical trial [30]. Naïve T cell depletion is also under investigation to decrease of chronic GVHD [31]. Besides ex vivo T cell depletion, in vivo depletion methods have also been employed using serotherapy as antithymocyte globulin (ATG) [32] or alemtuzumab [33]. Post-transplant high dose cyclophosphamide (PTCy) is another increasingly used methods in clinical practice in both adults and children that targets alloreactive T cells after T cell-replete HCT [11,34,35,36].

## 3. Rationale of αβ T Cell Depleted Hematopoietic Stem Cell Transplantation

Pre-clinical models of GVHD demonstrated that CD4+ and CD8+ T cells (=αβ T cells) to be major players in GVHD pathogenesis [37,38,39]. This causative correlation is the rationale for the use of αβ T cell depletion (rather than pan T cell depletion) allo HCT. The αβ T cell depletion is often combined with CD19+ B cell depletion for same reason explained above. The selective depletion of the αβ T cell from the infused graft spares γδ T cells and NK cells and likely favor their homeostatic reconstitution, thus potentially resulting in lower risk of infection [40,41] and relapse [42,43]. NK cells play a pivotal role in the defense against malignant transformed or virus-infected cells [44]. Allo-reactive NK cells have also been shown to positively affect the outcome of HCT via displaying GVT effect in children and adults without increasing risk of GVHD [8,45,46,47,48,49,50,51]. In murine models, NK allo-reactive cells were able to kill host dendritic cells (one of the antigen presenting cells = APCs), and this can contribute to reducing the risk of GVHD, since recipient APCs are known to play a major role in GVHD pathophysiology [52]. The γδ T cells population is a component of the innate immune system. They can directly recognize self-expressed stress-related (e.g., viral or oncogenic) antigen on the cell surface triggering immediate cytotoxic effect [53,54,55]. This is in distinction to the limited capability of the αβ T cells and NK cells that can only recognize MHC-related peptides of tumor-associated antigens. Several preclinical and clinical observations have suggested the antineoplastic effect of γδ T cell against hematological malignancies [56,57] and solid tumors [58,59]. These data have been corroborated in clinical studies showing improved relapse-free survival with higher post-transplant γδ T cell counts in the peripheral blood [42,60,61]. For example, one study has shown that higher γδ T cell (≥10% of total lymphocytes) in the peripheral blood in earlier post-transplant time (between 2–9 months) was an independent factor for improved DFS [61]. The γδ T cells, alike NK cells, have not been implicated in causing GVHD [62,63,64]. Moreover, the γδ T cells were shown to facilitate engraftment of allogeneic stem cells in preclinical models [65,66]. This favorable effect on engraftment was also suggested by clinical observation [67,68]. It is to be noted that despite the hypothesized favorable outcome of using αβ T cell depletion transplant, this approach was not directly compared to the traditional pan T cell depletion. Only Lang et al. [69] reported improved T and NK cell recovery following αβ T cell depletion transplant when compared to historical cases of pan T cell depletion.

## 4. Technical Methods

**Figure 2:**
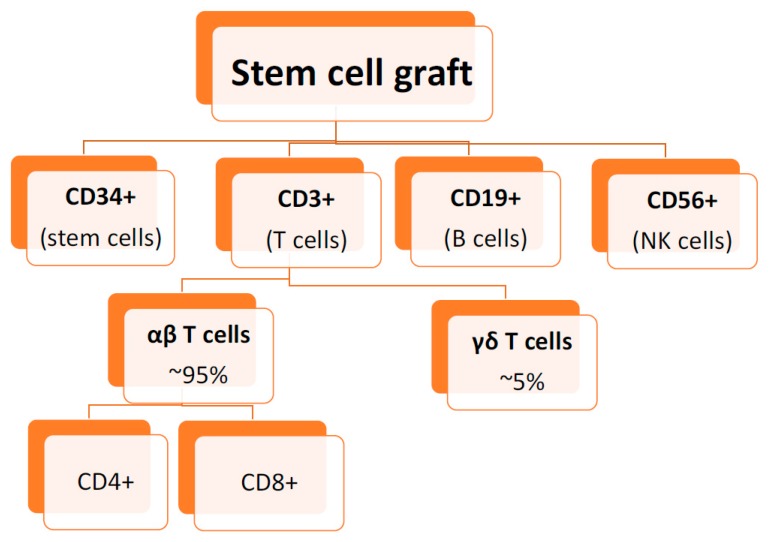
Key Component of the stem cell graft.

The HSC product contains a variety of cells including myeloid precursors and lymphocytes in addition to the minor component (~1%) of stem cells (Figure 2) [70]. Various methods have been employed for ex vivo T cell depletion and reviewed in recent literature [71]. The earliest clinical method involved the use of soybean lectin agglutination with T cell resetting with sheep red blood cells [72]. Subsequently, T cell monoclonal antibodies (in combination with immunotoxins or complement) were used [73]. The addition of complement or immunotoxins to the anti-T cell antibody is essential for elimination of the T cells. This was recognized after encountering high risk of GVHD with the earlier use of T cell monoclonal antibody alone [74]. The discovery of T10B9 by University of Kentucky (USA) allowed the selective depletion of the αβ TC [75]. The procedure of αβ T cell depletion has been described before [76,77,78]. An updated report of the procedure efficiency has also been published [76].

**Figure 3:**
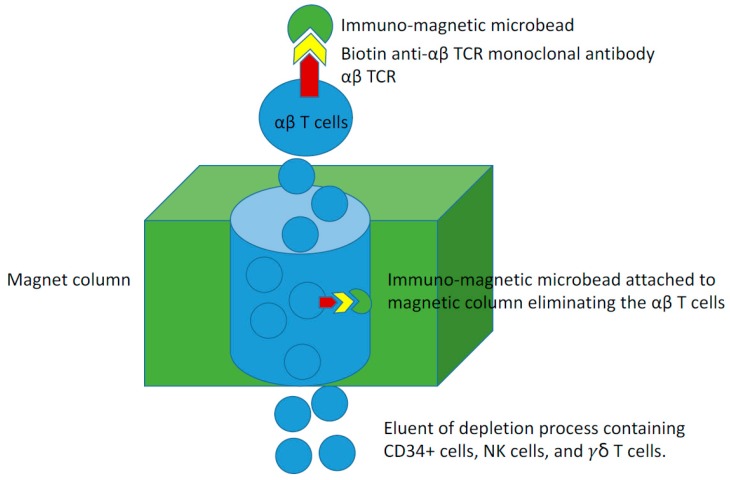
Immuno-magnetic microbead depletion process of the αβ T cells.

In summary, the graft processing for αβ T cell depletion is done using the CliniMACS device^®^ TCRαβ-Biotin system (Miltenyi Biotec, Bergisch Gladbach, Germany). The allogeneic donors are mobilized with filgrastim G-CSF for 4 days with leukapheresis starting on day 5 (and possibly day 6) per standard guidelines [79]. Peripheral blood CD34+ cell count is checked on the day of apheresis (day 5). A count of ≥ 40/µL is predictive of an adequate collection in one apheresis session, while a count < 20/µL often predict suboptimal collection (even in 2 sessions). In these donors, plerixafor is considered as described previously [80]. However, it is to be noted that plerixafor is not currently approved for this indication (volunteer donor) by the Food and Drug Administration in the USA. The target number of CD34+ stem cells in the apheresis product (i.e., prior to αβ T cell depletion) for pediatric population is 40 (minimum of 12–15) × 10^6^ cells/kg recipient weight. The leukapheresis product then undergoes negative selection (i.e., depletion) of the αβ T cells prior to infusion to the patient. This depletion typically results in ~20 (minimum of 8–10) × 10^6^ cells/kg CD34+ cells (i.e., allowing for up to 40% loss during the depletion procedure). Prior to the immunomagnetic labeling of the apheresis product, it is washed to remove platelets and the cell concentration will be adjusted in preparation for antibody labeling. The apheresis product is then labeled using the CliniMACS TCRαβ Biotin kit (Miltenyi Biotec, Bergisch Gladbach, Germany) and CD19+ immunomagnetic microbeads. After immunomagnetic labeling, the cells are washed to remove unbound microbeads (Figure 3). The labeled product is loaded onto the CliniMACS device where labeled cells are depleted and the negative fraction is eluted off the device. This negative fraction is then centrifuged and volume- reconstituted to obtain the final product. We do not have a maximum limit of CD34+ cells to be infused, however, we target a maximum dose of αβ T cells of 1 × 10^5^/kg at the end of the negative depletion procedure. If the residual number of αβ T cells is >1 × 10^5^/kg, a selected part of the product can be eliminated and cryopreserved. If this exclusion compromises the minimum CD34+ stem cells, we perform CD34+ cell selection on that part of the graft. Our transplant protocol typically involves using rituximab at day +1 to eradicate residual B cell in the product unless the CD19+ B cells in the final product is <1 × 10^5^ CD19+ cells/kg.

We sometimes use BM product if the donor is a child or an adult donor declines PBSC apheresis. However, it is to be noted that, in order to optimize the selection process, the maximum volume of packed red blood cells (RBCs) in the product (pre-selection) that is allowed to go on the CliniMACS column is 30 mL (i.e., 100 mL of BM with 30% HCT). Therefore, BM product is RBC-depleted using Ficoll^®^ density gradient separation (GE Healthcare Bio-Sciences, Pittsburgh, PA, USA) prior to proceeding with selection on the CliniMACS device. The RBC-reduced product is stored at +1 to +8 °C until used.

## 5. Clinical Outcome of AB T Cells Depletion HCT

The utilization of αβ T cells depletion in allogenic HCT has been evaluated in treating both malignant and non-malignant etiologies [81]. The majority of the published studies have been conducted in pediatric haplo HCT setting [61,82]. Selected seven studies are summarized and discussed below (Table 1) [69,83,84,85,86,87,88]. Transplant outcome using this approach has also been described in other studies [28,29,82]. Several other individual case reports have reported the use of αβ T cells depletion in different non-malignant conditions as Wiskott–Aldrich, β thalassemia and Hoyeraal-Hreidarsson syndrome with favorable outcome [89,90,91].

## 6. Conditioning Regimens and CD34+ Cell Dose

Conditioning regimens used in these studies were either myeloablative [69,87,88] or reduced intensity [83,84,85,86]. The majority of patients in these studies received haplo HCT except for matched unrelated donors used in two studies [84,85]. The median dose of CD 34+ cells ranged from 12 to 16 × 10^6^/kg with median residual αβ T cells dose of 1 to 4 × 10^4^/kg.

## 7. Engraftment and Immune Reconstitution

The engraftment failure rate was variable among different studies ranging from 0% reported by Maschan et al. [85], and up to 27% by Balashov et al. [84]. The median day for neutrophil and platelet count recovery reported in these studies ranged from 12–16 and 10–14 days respectively. The recovery of γδ T cells preceded αβ T cells with a median of 7–10 days. Two studies reported similar results of immune reconstitution with T cell >500/µL and B cell >200/µL on day +120 [69,84]. Another study reported similar data for T cell recovery at day 120 but delayed B cell recovery >150/µL until 6 months [83].

## 8. Graft Versus Host Disease (GVHD)

Different studies used different prophylaxis regimens against GVHD. Antithymocyte globulin (ATG) was used in all studies as part of the preparative regimen. Only one study used OKT3 instead of ATG before 2012 in a subset of 34 patients [69]. While a German group [69] used ATG distal to day 0 (day −9 to −12) with the primary purpose of prevention of graft failure, an Italian group [83] used it more proximal to day 0 (day −3 to −5) in order to prevent both graft failure and GVHD hypothesizing that this will not influence the post-transplant recovery of γδ T cells which is expected to occur after biological elimination of ATG. No pharmacologic GVHD prophylaxis was used by an Italian study [83]. Three studies used single agent mycophenolate mofetil [69,86,87] and another study used tacrolimus in all patients plus methotrexate (*n* = 34), mycophenolate mofetil (*n* = 2), or cyclosporine (*n* = 1) [84]. In another study, single agent tacrolimus (*n* = 2), methotrexate (*n* = 5) or both (*n* = 21) were used while 5 patients did not receive pharmacologic prophylaxis [85]. B cell depletion was done simultaneously in most studies via ex vivo CD 19 depletion (along with αβ T cell depletion). Two studies used rituximab for in vivo B-cell depletion [83,84].

The rate of acute and chronic GVHD (aGVHD and cGVHD) was variable among studies, but occurred at low rate and was mostly low grade. The lowest incidence of aGVHD of 3% reported by one study [86]. Bertaina et al reported 13% risk of aGVHD (mostly grade I-II and only skin involvement) with no reported cGVHD with a median follow-up of 18 months [83]. In another study, the risk of aGVHD was 25% (with 15% risk of grade III) with a cGVHD risk of 27% (extensive disease of 9%) [69]. In the adult cohort by Kaynar et al. 38% developed aGVHD (grade I–II was 27%), and 6% developed cGVHD (2 patients; one was extensive) [87]. In the study by Balashov et al. 23% developed aGVHD (with one patient with grade IV that turned into refractory cGVHD) [84]. The highest incidence of grade II-III aGVHD was reported by Maschan et al. as 39% (none developed grade IV) with risk of cGVHD of 30% with a median follow up of 2 years (some patients received donor lymphocyte infusion) [85]. In the report by Gonzalez et al. risk of aGVHD and cGVHD was 18% and 14% respectively [88].

## 9. Infections

The incidence of CMV reactivation ranged from 23–74%. Death due to CMV was reported in 1 case (4%) by Bertaina et al. and 2 cases (6%) by Maschan et al. [83,85]. Many studies used rituximab for CD19+ B cell depletion to mitigate the risk of EBV reactivation. Only Maschan et al. reported significant EBV reactivation (50%) [87]. BK viremia was reported in two studies at a rate of 16% and 25% [86,87].

## 10. Relapse and Survival

The relapse of malignancy post-transplant was the major cause of mortality. Relapse rates are ranging from 22–58% while relapse-related mortality rates 19–41%. As expected, the OS was discrepant between patients with malignant and non-malignant diseases. For non-malignant diseases OS reported by Bertaina et al. and Balashov et al. at 92% and 97% respectively [83,84]. For malignant diseases, Kaynar et al., Lang et al. and Maschan et al. reported lower OS rates of 54%, 51% and 67% [69,85,87].

## 11. Conclusions and Future Perspective

These clinical data are suggestive of a promising role of αβ T cell depletion to overcome the HLA disparity haplo HCT. Although this approach is adopted by several European centers, it has not gotten a wide utilization in the USA except for few pediatric centers. Several ongoing studies are under way using either haplo or HLA-matched HCT (Table 2). Comparative studies are lacking to compare αβ T cell depletion HCT and other modalities of haplo HCT such as Pan T cell depletion or PTCy. Clinical trials evaluating the therapeutic utility of γδ T cells for hematological malignancies are lacking. An ongoing phase I trial is underway to evaluate the safety and feasibility of infusing add-back αβ T cell-depleted product after haplo HCT (NCT02193880). Suicide gene (caspase-9) programming of the add-back T cells has been used in order to eliminate the T cells (via therapeutic activation of the suicide gene) in case severe GVHD develops [92]. This approach is currently under investigation (NCT01744223).

## Figures and Tables

**Table 1:** Outcome of selected clinical studies using αβ T cell/CD19+ B cell depletion HCT. Only one study (Kaynar et al.) performed αβ T cell depletion without CD19+ B cell depletion.

Study	Bertaina et al. [83]	Balashov et al. [84]	Lang et al. [69]	Maschan et al. [85]	Lang et al. [86]	Kaynar et al. [87] (no CD19 depletion)	Gonzalez et al. [88]
Year published	2014	2015	2015	2016	2016	2017	2017
Number of patients	23	37	41	33	30	34	27
Age group	Children	Children	Children	Children	Children	Adult	Children
Disease	Non-malignant	Primary immunodeficiency syndromes	AML, MDS, and non malignant	AML	ALL, AML, solid tumors, non malignant	AML and ALL	AML
Study design	Prospective	Prospective	Retrospective	Prospective	Prospective	Retrospective	Retrospective
Conditioning regimen	RIC	RIC	MA	RIC	RIC	MA	MA
CD34^+^ cell dose per KG	15.8 × 10^6^	11.7 × 10^6^	14.9 × 10^6^	NA	14.6 × 10^6^	12.69 × 10^6^	6.41 × 10^6^
αβ CD3^+^ T cells dose per kg	4 × 10^4^	10.6 × 10^3^	16.9 × 10^3^	NA	14 × 10^3^	11.72 × 10^3^	11 × 10^4^
ANC recovery day	13	16	10	16	12	12	13
platelet recovery day	10	NR	NR	14	15	11	10
Graft failure (primary/secondary)	16%	27%	12%	0%	23%	17%	3.7%
Acute GVHD II-IV risk	13% (no G III–IV)	24%	25%	39%	3%	38% (all grades)	18% (III–IV)
Chronic GVHD risk	0% (at 18 months)	5%	27%	30%	NA	6%	14%
CMV reactivation	38% (including adenovirus)	46%	NA	52%	23%	73.5%	NA
EBV reactivation	NR	NR	NR	50%	0%	0%	NR
BK virus reactivation	NR	NR	NR	NR	16%	25%	NR
NRM	9%	3%	9.7%	10%	3%	20%	18.5%
Relapse	9%	3%	47%	30%	3% (at 100 days)	58%	22%
Survival	91% (2-year OS)	97% (1-year OS)	51% (1-year OS)	67% (2-year OS)	94% (100-day OS)	54% (1-year OS)	62% (18-month OS)

ALL = acute lymphoblastic leukemia, AML = acute myeloid leukemia, ANC = absolute neutrophil count, CMV = cytomegalovirus, EBV = Epstein Barr virus, GVHD = graft versus host disease, MDS = myelodysplastic syndrome, NA = not applicable, DFS = disease-free survival, MA = myeloablative, NR, not reported, NRM = non-relapse mortality, OS = overall survival, RIC = reduced intensity regimen. Shaded columns indicates studies of non-malignant disorders.

**Table 2:** Selected ongoing clinical trials of αβ T cell depletion (all are pediatric studies).

Trial	Disease	Donor	Country	Phase
NCT02327351	Primary Immunodeficiency	MUD/Haplo	Russia	II/III
NCT01810120	Malignant/Non-malignant	Haplo	Italy	I/II
NCT02065869	Malignant/Non-malignant	Haplo	USA-Bellicum	I/II
NCT02508038	Malignant	Haplo	USA-Wisconsin	I
NCT02600208	Leukemias/lymphomas	MUD/Haplo	USA-Wisconsin	II/III
NCT02990819	Primary Immunodeficiency	MUD/Haplo	USA-Philadelphia	II
NCT03047746	Bone marrow failure	MUD/Haplo	USA-Philadelphia	I

MUD = HLA-matched unrelated donor.
